# The Emerging Role of Succinate Dehyrogenase Genes (SDHx) in Tumorigenesis 

**Published:** 2019-04-01

**Authors:** Elham Nazar, Fatemeh Khatami, Hiva Saffar, Seyed Mohammad Tavangar

**Affiliations:** 1 Department of Pathology, Shariati Hospital, Tehran University of Medical Sciences, Tehran, Iran; 2 Chronic Diseases Research Center, Endocrinology and Metabolism Population Sciences Institute, Tehran University of Medical Sciences, Tehran, Iran

**Keywords:** Succinate dehydrogenases, Tumor, Genetic

## Abstract

Transformation of a normal cell to cancerous one is dependent on the accumulation of several genetic and epigenetic alterations. One of the candidate driver genetic alterations can happen in succinate dehydrogenases (SDHx) coding gene include SDHA, SDHB, SDHC, SDHD, and SDHAF2. The most important SDH mutation is in the SDHD gene, which encodes the smallest subunit of mitochondrial complex II (SDH). It has key function both in familial and non-familial hereditary paraganglioma/phaeochromocytoma syndrome (HPGL/PCC). SDHx genes mutations can have resulted in genetic and epigenetic changes like histone hypermethylation. These properties can lead to succinate-mediated inhibition of α-ketoglutarate-dependent dioxygenases. So hypoxic conditions can generate subsequent neoplastic transformation, and in this review, we are presenting the role of SDHx in several malignancies.

## Introduction

 Tumorigenesis is a multistep process depending on a sequential accumulation of genetic and epigenetic alterations within the cells ^1^^,^^2^ . In spite of the fact that during tumorigenesis a large number of mutations are involved, only a relatively small subset of driver mutations is crucial for starting steps of neoplastic development. These mutations consequence in destruction of tissue homeostasis as the transformed cells gain fitness by increasing their proliferation rate, decreasing their death rate, and creating a growth-promoting environment^3^. Histological studies and Immunohistochemistry (IHC) are applicable for the tissue distribution of targeted antigens in the way of neoplastic and non-neoplastic diagnosis because specific antigens are expressed de novo or up-regulated in certain lesions^4^^-^^8^.  One of the remarkable of these driver genetic alterations can happen in succinate dehyrogenase genes (SDHx). SDHx is a multipart enzyme made of subunits encoded by SDHA, SDHB, SDHC and SDHD genes. Succinate dehydrogenase (SDH) heterotetrameric complex catalyzes the oxidation of succinate to fumarate in the tricarboxylic acid (TCA) cycle and in the aerobic respiratory sequences of eukaryotes and bacteria^9^. Succinate dehydrogenase and fumarate hydratase (other enzyme of Krebs cycle) inactivation result in an obstruction of Krebs cycle, impaired respiration and abnormal accretion of their substrates, succinate and fumarate^10^. Krebs cycle genes, fumarate hydratase (FH) and SDH are mutated in a compartment of several malignancies, secondary to accretion of their substrates, fumarate and succinate, respectively^11^. SDH enzyme (also known as succinate-ubiquinone oxydoreductase) is a well-preserved heterotetrameric protein, with SDHA and SDHB as catalytic subunits, which is produced in the mitochondrial matrix and anchored to the inner membrane^12^. The results of inactivation of SDH and FH have both been associated with abnormalities of cellular metabolism, responsible for the activation of hypoxic gene response pathways and epigenetic alterations (eg, DNA methylation) ^13^. Loss of the SDH complex is described in extra-adrenal paragangliomas, gastrointestinal stromal tumors, renal cell carcinomas and rare in other epithelial tumors^14^. Germline mutations in FH gene influence individuals with leiomyomas and renal cell cancer (HLRCC), while mutations in SDH can be the cause of paragangliomas and phaeochromocytomas (endocrine tumors)^15^. Paragangliomas are neural crest-derived tumors that begin from parasympathetic ganglia of the head and neck areas or from sympathetic ganglia sited in the thorax, abdomen or pelvis. These tumors may grow in the adrenal medulla, in which case, they are called pheochromocytomas^16^. Gastrointestinal stromal tumors (GISTs) are infrequent mesenchymal tumors of the GI tract. These tumors start in very early forms of special cells in the wall of the GI tract named the interstitial cells of Cajal (ICCs)^17^^,^^18^. Gastrointestinal stromal tumor (GIST) and paraganglioma are caused by germline mutations in SDH subunits B, C or D^   19 ^. Because of their strong syndromic and heritable source and distinctive history, SDH-deficient tumors are essential to be identified^20^. As a rule, it is suggested that genetic testing for SDHx which could be SDHA, SDHB, SDHC and SDHD be available each time an SDH-deficient tumor is observed^21^. In this review article, several types of SDH deficient tumors are focused on.

**SDH mutations**

**Figure 1:**
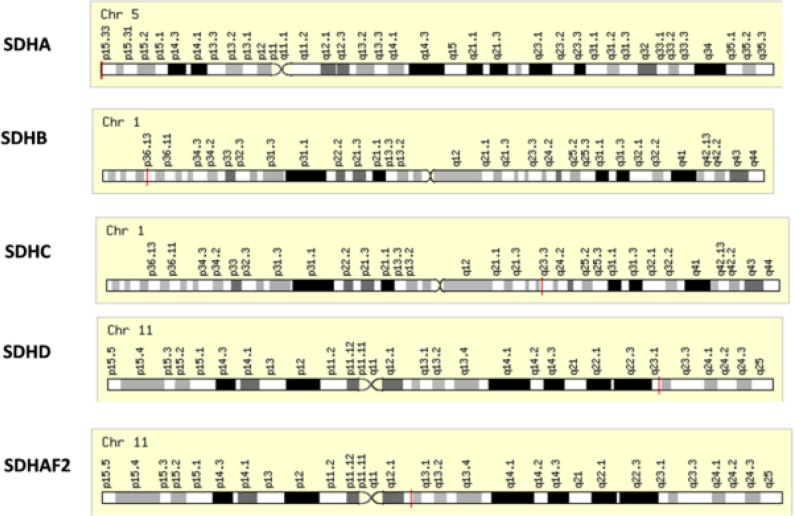
The genomic location of several Succinate Dehydrogenase Complex Subunits: bands according to Ensemble, locations according to GeneLoc (and/or Entrez Gene and/or Ensembl if different).

Mitochondrial DNA mutations have been found in dissimilar cancers and seem to change mitochondrial metabolism, increase risk of tumorigenesis and allow cancer cell modification to changing environments^22^. The nuclear-encoded Krebs cycle enzymes in the mitochondria, fumarate hydratase (FH) and succinate dehydrogenase (SDHB, -C and -D) act as tumor suppressors^15^. Although mutations in all subunits occur in cancer, tumors containing mutations in the catalytic subunit SDHB are predominantly malignant and associated with enhanced risk of metastasis^10^. SDHD, SDHB and SDHC mutations are a basis for a series of molecular procedures leading to the abnormal stabilization of hypoxia-inducible factors (HIF) under normoxic or hypoxic conditions or pseudo-hypoxia (via inactivation of SDH, accumulation of succinate, inhibition of prolyl-4-hydroxylases and subsequent HIF hydroxylation), thus, encouraging cell proliferation, angiogenesis and tumor genesis ^23^. SDH deficiency reduces prolyl hydroxylases and hydroxylation of hypoxia-inducible factor-α, resulting in activation of the hypoxia pathway, angiogenesis, glucose metabolism, cell motility and cancer cells survival ^24^^,^^25^. Although most of the oncogenic activities of SDH mutations have been known to be related to a metabolite, succinate, which accumulates in SDH-deficient cells^26^, in another way, these cells show high levels of succinate also accessible with elevated caspase 3 and/or caspase 7 levels^27^. Other subunits of SDH include succinate dehydrogenase complex assembly factor 1 (SDHAF1), which is a novel LYR-motif protein; the first SDH assembly factor recognized in any organism, and is found within the mitochondrial matrix and the succinate dehydrogenase complex assembly factor 2 (SDHAF2) which is shown to be significant for the acceptable flavination of SDHA and function of the SDH complex^28^. The SDH genes located on chromosomes 1, 5 and 11 (Figure 1) encode subunits of the heterotetrameric succinate dehydrogenase complex, an element of both the mitochondrial-respiratory chain (complex II) and the Krebs cycle in which SDHA (Ch5p15) and SDHB (Ch1p36) encode the two catalytic subunits, the flavoprotein and the iron-sulfur protein, respectively. SDHC (Ch1q21) and SDHD (Ch11q23) encode transmembrane proteins that anchor complex II in the inner mitochondrial membrane, and include a ubiquinone binding site^29^^.^

Genetic analysis showed a novel frame shift SDHD mutation resulting in premature stop codon at amino acids 133 of the protein^30^. Finally, the combination of loss-of-function germline mutations in one of the SDH subunit genes and somatic loss-of-function mutations in the tumor cells resulted in inactivation of both alleles. In some cases, the mechanism of SDH inactivation is indistinct and is probably related to epigenetic silencing ^31^. Negative immunohistochemistry (IHC) staining for SDHB is linked with the SDH mutations (SDHB, SDHC or SDHD) that can weaken the whole enzyme complex activity^4^^,^^32^. SDHB immune staining was extremely concordant with the immunoblot finding^33^. Totally, lack of staining is more generally found with SDHB mutation, contrary to weak diffuse staining often linked with SDHD mutation^34^. SDHA and SDHB IHC must be interpreted with caution and potential false-positive or false-negative results should be considered; some supporting results can be provided by molecular testing. For example, in SDHD mutation, weak non-specific cytoplasmic staining usually occurs, and this pattern of staining can be difficult to interpret with confidence^35^. Also, pseudo-hypoxia, the major phenomenon shown to date to mediate the tumorigenic ending of the loss of mitochondrial tumor suppressors, is a common mechanism for both SDH and FH mutations and in some tumors with SDHD or SDHB mutations, the hypoxia-inducible factor (HIF) pathway, and therefore an angiogenic reaction are activated due to high vascular density^36^. SDH mutation due to activation of the hypoxia pathway, supports tumor formation by activating angiogenesis, glucose metabolism, cell motility and cell survival^24^^.^ Activation of the HIF pathway in SDH may be dependable on the stimulation of glycolysis and anaerobic fermentation^37^^.^ Thus, SDHB and SDHD inactivation is associated with deregulation of the HIF-1 and HIF-2 transcription factors, and a non-HIF-dependent pathway involving JunB, cJun and EglN3/PHD3 in normal developmental apoptosis in sympathetic neuronal progenitor cells^38^. Also, the loss of SDH mutation causes succinate accretion and reduces α-ketoglutarate-dependent dioxygenase enzymes such as the TET family of DNA hydroxylases. TET proteins catalyze the alteration of 5-methylcytosine to 5-hydroxymethyl cytosine (5-hmC), which is essential for consequent DNA demethylation^39^. Therefore, SDH and FH mutations can inhibit DNA and histone demethylases, leading to loss of 5hmC and low level 5hmC in SDH-deficient tumors associated notably with nuclear exclusion of TET protein. The results are inhibition of the TET family of DNA hydroxylases, increased succinate and fumarate which negatively affect the enzyme activity of histone demethylases^40^. Also, SDHD mutation has a distinctive phenotype and recognized increased age-related tumor risks with extremely destabilizing SDHB missense mutations ^41^. Therefore, the germline mutations could suggest that these patients should be considered for the risk of progression of other cancers ^42^. A possible advantage in identifying metabolic-enzyme mutations that are pathogenic in specific cancers is that such cancers may be susceptible to pharmacologic administration that are more effective and less toxic than obtainable therapies^43^. The inhibition of these molecular pathways portrays the widespread vascularization of SDH-related tumors which may be due to metastatic spreading by driving epithelial-to-mesenchymal transition in SDHB-deficient tumors^44^. Another significant result including SDHA is lost collectively with SDHB in SDHA-mutated tumors, but its expression remains in tumors with other SDH mutations^        35 ^. Thus, genetic testing allows familial consultation and identifies persons at high risk of malignancy (SDHB mutations) or considerable multiorgan disease^34^.

Paraganglioma (PGL) and pheocromocytoma (PCC)

Pheochromocytomas and paragangliomas are rare diseases, but frequently occur with nonspecific symptoms ^45^^-^^48^ . Pheochromocytomas and paragangliomas are tumors beginning from the adrenal medulla and sympathetic/parasympathetic paraganglia, respectively^49^. The most frequent location of paragangliomas is the carotid body^50^. Symptoms of this tumors consist of increased blood pressure, headache, sweating and palpitations^51^. The diagnosis is typically established by calculating the level of catecholamines or their metabolites in urine or plasma, and also single consistent complete sign of malignancy in pheochromocytoma is the presence of metastasis^52^. Positive staining for chromogranin and synaptophysin is present in the chief cells, whereas the sustentacular cells are positive to S100 protein in immunohistochemistry staining ^53^^,^^54^.

 Multifocal tumors, young age and positive family history, known features related to inheritance are not present in all patients, which leads to important study on considerable genetic mutations results^55^. According to genetic study results, it has been recommended that negative immunostaining of SDHB can be taken as a marker for the presence of a mutation in one of the five SDH genes^48^^,^^56^. Recognition of patients with inherited pheochromocytoma is significant because it can guide medical administration in mutation-positive patients and their families^        57 ^. Pheochromocytomas and paragangliomas are neuroendocrine tumors that occur sporadically and in some heritable tumor syndromes due to germline mutations in SDHB, SDHC or SDHD genes^58^. Three other important hereditary familial cancer syndromes [von Hippel-Lindau (VHL) disease, multiple endocrine neoplasia (MEN) types 2A and 2B, and neurofibromatosis type 1] are also associated with PCC susceptibility^38^^,^^59^. The benefit of evaluation by IHC and the possible advantages of Ki67 antigen, c-erbB-2 and c-kit proto-oncogenes in the discrimination of benign and malignant pheochromocytomas were reported^60^. However, there is a widespread genetic description for PGL/PCC^61^. At least, there is germline mutation in one of the ten recognized susceptibility genes: RET, NF1, VHL, SDHAF2, TMEM127 or MAX, and in genes encoding the four subunits of succinate dehydrogenase (SDHA, SDHB, SDHC or SDHD, referred to as SDHx genes) associated with PGL/PCC. In all SDHD and SDHC cases, but not SDHB tumors, these were found in the head and neck areas^62^. These syndromes affect mutations in one of the three subunits of the SDH gene. By way of illustration, type 1 is associated with SDHD, type 2 is associated with an unknown gene, type 3 is associated with SDHC and type 4 with SDHB^63^. Analysis of SDHD can also help to discriminate synchronous primary tumors from abdominal metastases ^64^. Also, SDHD are more possible to have multifocal disease when compared with patients with SDHB and SDHC mutations ^65^. SDHB-mutation carriers have higher risk of developing a metastatic disease and shorter survival than patients with a malignant PGL/PCC but without SDHB mutations^16^. Thus, young age and metastatic disease are both factors for SDHB mutation^66^. Altogether, extra-adrenal sympathetic tumors are commonly related to SDHB (predominantly solitary, large tumors), less often to SDHD, infrequently to SDHC and SDHA mutations, and because of this connection if SDHD, SDHB and SDHC testing have negative results, then SDHAF2 mutation should be checked^48^. The SDHAF2 gene encodes an SDH co-factor related to the role of the SDHA subunit and is currently entirely related with head and neck paragangliomas ^28^. Patients with SDHB mutations are younger, more commonly have extra-adrenal tumors and a shorter metanephrine excretion doubling time-related to shorter survival^67^. On the whole, SDHB immunohistochemistry on pheochromocytomas and paragangliomas could develop the diagnosis of pheochromocytoma-paraganglioma syndrome^68^. Also, SDHx-PPGLs overexpress somatostatin receptors (SSTRs) and are consequently targetable with somatostatin analogs (SSAs) labeled with diagnostic radionuclides ^69^.

**Gastrointestinal stromal tumor (GISTs)**

The interstitial cells of Cajal are origin site in which GIST arise depending on high-level KIT expression for lineage specification and survival. Majority of the sporadic GISTs harbor activating mutations in KIT and to a lesser extent, in PDGFRA and BRAF^70^. Genetic mutations affecting KIT, PDGFRA, BRAF and SDH complex functions are thought to be mutually special events^71^. GIST diagnosis is based on histology changes in tissue sections, but not clinical symptoms. While spindle and/or epithelioid tumor cells in the gastrointestinal tract are positive for KIT or DOG1 in immunostaining, GIST could be considered. DOG1 is more specific for GIST than KIT and is occasionally positive for KIT-negative GIST. Epithelioid tumor cells may be a definite type of GIST, including PDGFRA-mutated GIST or GIST with mutations in the SDH complex, or GIST transformed to highly malignant (typically mixed phenotype)^72^. Thus, patients accessible to generally epithelioid GISTs were characterized by plump cells containing a centrally located, round nucleus and prominent nucleoli; these changes were approximately distinguishable from those seen in patients with SDH mutated GIST^73^. Also, among GISTs that begin in children and young adults, insulin-like growth factor 1 receptor (IGF1R) overexpression is usually observed in those with KIT/PDGFRA wild-type but not in those with either mutant kinase^   74 ^. This result which leads to IFG1R-positivity may also be a helpful serology marker to recognize SDH-deficient GISTs^75^. SDHB and SDHA mutated GIST consist of a subgroup of young adult women patients with a well distinct clinical and biological profile, usually characterized by the gastric primary tumor localization, a principally mixed epithelioid and spindle cell morphology, diffused IHC positivity for KIT and revealed on gastrointestinal stromal tumors 1 (DOG1), recurrent lymph node metastases, and an nonaggressive course of disease even if metastasis is identified. Moreover, they are distinguished by the overexpression of the insulin growth factor 1 receptor (IGF1R). GIST characterized by SDHB, −C or D mutations (most of them germline, and in few cases by SDHA mutations), originate mainly from the stomach, with a lesser female incidence, but histologically like SDHA mutated GIST^76^. In pediatric patients, mostly GISTs are KIT/PDGFRA wild-type. These findings are principally on girls and usually have a clinically slow progress course7^77^. Also, these tumors did not reveal the KIT or platelet-derived growth factor receptor-α (PDGFRA) gene mutations related to GISTs and correlated lesions that are responsive to Imatinib mesylate and its analogs^78^. The most common and closest change detected by tumor genetic studies is the deletion of the 1cen-q21 chromosomal region involving the SDHC gene. An additional change was also discovered, together with loss of the 1p region^79^. Not more than a small subset of SDHB-deficient GISTs carries loss-of-function mutations in SDHB, SDHC or SDHD. Because of the complication of its locus (15 exons) and the presence of three pseudogenes, SDHA is infrequently analyzed. Moreover, immunohistochemistry for SDHA can be used to select patients for SDHA-specific genetic testing^80^. Carney-Stratakis syndrome can make patients susceptible to GIST and paraganglioma ^19^. Carney triad (CT) shows the association of paragangliomas (PGLs) with GISTs and pulmonary chondromas in which inactivating mutations of the mitochondrial complex II SDH enzyme subunits SDHB, SDHC and SDHD are found in PGLs, gain-of-function mutations of c-kit (KIT), and platelet-derived growth factor receptor A (PDGFRA) in GISTs^81^. Also, those with SDHA-negative GISTs had older median age, lower female to male ratio but like mitotic counts and median tumor sizes, with a slow course of disease in most cases, regardless of a vaguely higher rate of liver metastases^82^. Patients with metastatic KIT/PDGFRA wild-type succinate dehydrogenase–deficient gastrointestinal stromal tumors harboring succinate dehydrogenase subunit A mutations show a remarkable long survival. These patients should be recognized in clinical practice to improve altered treatments and follow-up over time^83^. Furthermore, patients with SDHX mutations need germline testing to establish whether the mutation is sporadic or germline, and if a germline mutation is shown, genetic consultation is indicated. In contrast, those patients found to have SDHC promoter hypermethylation do not require genetic consultation, as these are not germline alterations. However, these patients still do need screening for paragangliomas as noted, since they are often related to syndromic GIST^84^.

**Renal cell carcinoma (RCC)**

Renal cell carcinoma (RCC) originates from the kidney and is frequently present with only some symptoms, but it can have varied paraneoplastic appearances. It shows relative resistance to radiation and chemotherapy and the latest study on the genetic changes related to RCC have shown new targets for therapy^   85 ^. SDH-deficient renal carcinoma has been established as a conditional entity in the 2013 International Society of Urological Pathology Vancouver Classification^86^. SDH-deficient renal carcinoma is recently accepted under the World Health Organization (WHO) 2016 classification and presents vacuolated eosinophilic cytoplasmic and cytoplasmic inclusions. It is predominantly related to SDHB mutation, although SDHC and SDHA mutations can occur^87^. Thus, the 2016 World Health Organization (WHO) classification includes new renal tumor classification; recently accepted epithelial renal tumors are hereditary leiomyomatosis and renal cell carcinoma (RCC) syndrome–associated RCC, succinate dehydrogenase–deficient RCC, tubulocystic RCC, acquired cystic disease–associated RCC, and clear cell papillary RCC^88^. Moreover, germline mutations in Krebs cycle enzyme, SDH, can also cause a hereditary tumor susceptibility syndrome^89^^.^ Most SDH-deficient tumors contain SDHB mutation, with only a small number of RCC with SDHC or SDHD having been reported to date^90^. Thus, a monomorphic oncocytic renal tumor with a solid architecture, cytoplasmic inclusions of flocculent material, and intratumoral mast cells should be quickly assessed for SDH status, as it may have indications for screening the patient and families. In this manner, negative IHC for KIT and heterogeneous staining for epithelial antigens are other helpful approaches^91^. Also, mitochondrial dysfunction may lead to neoplasia through the role of mitochondria in apoptosis. Because RCCs are referred to as “oncocytic” (that is, accumulation with mitochondria), the role of SDH in RCC is reasonable^92^. 

However, renal cell carcinomas (RCCs) with other histologic features have been reported in patients with germline mutations of SDH subunit genes and a few RCCs of other histologic types have been established to be SDH-deficient with the lack of identified germline gene mutation^91^. The tumor had a mixed histology pattern of high-grade papillary and collecting duct carcinoma and characteristic pale eosinophilic cytoplasmic inclusions like SDHB-deficient RCC; this is the first report that identifies SDHA inactivation in RCC^93^. Despite the fact that most tumors are low grade, a lot of other tumors can be shown in an aggressive fashion, predominantly if they are high nuclear grade, and have coagulative necrosis or sarcomatoid differentiation ^        94 ^. Moreover, these suggested careful observations of patients at risk of SDH mutation related renal cell carcinoma and extensive surgical excision of renal tumors^   95 ^ and following the genetic consequences, a succession of tyrosine kinase inhibitors was administered as targeted treatment options and obviously showed how the genetic findings make the accessible motivation for their helpfulness^96^.

**Other epithelial tumors**

The succinate dehydrogenase complex catalyzes the oxidation of succinate to fumarate; mutations in its subunits SDHA, SDHB, SDHC and SDHD, and in the assembly factor SDHAF2, result in syndromes with divergent tumor types, including pheochromocytoma/paraganglioma, gastrointestinal stromal tumor, and, less often, renal-cell carcinoma, pituitary adenoma^24^ ,and breast cancer^26^. Also, head and neck PGLs, extra-adrenal PGLs, a number of other neuroendocrine or non-neuroendocrine neoplasms have been related to mutations in SDH genes. Thyroid cancer is the most common endocrine tumor in which SDHB and SDHD mutations are linked to epigenetic alterations^97^. A distinctive case of testicular seminoma has been reported in a carrier of germline SDHD mutations, which presented the loss of the wild type allele in tumor cells. The frequent neural crest embryonal origin of both pheochromocytoma and neuroblastoma with the common loss of the locus 1p35-36 in the last tumors, a section where SDHB gene is located, suggested that genetic alterations in SDHB might be related to the progression or advanced neuroblastoma tumors^12^. Papillary thyroid cancer from SDHx mutation is also seen in cowden syndrome^98^. The homozygous/compound heterozygous mutations in SDHA result in rigorous neurological dysfunction and cardiomyopathy, but heterozygous germline mutations in SDHB-D cause a pheochromocytoma-PGL syndrome. Some carriers of SDHB or SDHD mutations have been found to have renal cell carcinoma or papillary thyroid cancer which also appears in Cowden syndrome^99^. However, mutations in SDHA cause the lethal pediatric neurodegenerative disease and Leigh's syndrome but not paraganglioma^100^. In Leigh syndrome, a severe neurodegenerative disease, there was a compound heterozygous germline SDHA-mutation (one allele with nonsense and another with missense mutation) ^82^. Thus, germline SDHA mutations are related to juvenile encephalopathy ^   101 ^. Also, several other neoplasms have been reported in SDHx mutation carriers, consisting of pancreatic neuroendocrine tumor, adrenal cortical adenoma, neuroblastoma, ganglioneuroma, adenomatoid tumor of the adrenal gland, melanoma, lung cancer, breast carcinoma, esophageal cancer, rectal and ovarian carcinomas, uterine adenocarcinoma, uterine leiomyoma, testicular seminoma, bladder cancer, meningioma, oligodendroglioma, cecal polyps and hematolymphoid malignancies^   102 ^^.^ Moreover, decreased SDH enzymatic activity supports SDHD's involvement in the pituitary tumor development, testicular seminoma and papillary thyroid carcinoma^30^. Another finding shows that SDH mutation-related pituitary tumors have an aggressive phenotype^103^ and can be larger and further expected to produce prolactin than other pituitary adenomas^104^. A pancreatic neuroendocrine tumor is also within the SDH-related tumor variety^105^^,^^106^.

## CONCLUSION

 In total, the SDH-germline mutation can be driver mutations of Paraganglioma, Pheochromocytoma, Gastrointestinal stromal tumors, Renal Cell carcinomas and other endocrine-related tumors. The most important gene of SDH enzymatic complex is SDHB. Both genetic alterations and epigenetic changes (methylation) of this gene are considered as an important modification in endocrine malignancies formation. The bi-allelic expression of *SDHD* and the complete loss of SDH enzymatic activity whenever one of its subunits is mutated can describe the phenotypic variability in some tumors.
